# Insurance Status Is Related to Receipt of Therapy and Survival in Patients with Early-Stage Pancreatic Exocrine Carcinoma

**DOI:** 10.1155/2017/4354592

**Published:** 2017-04-10

**Authors:** Emily Boevers, Bradley D. McDowell, Sarah L. Mott, Anna M. Button, Charles F. Lynch

**Affiliations:** ^1^Carver College of Medicine, University of Iowa, Iowa City, IA 52242, USA; ^2^Holden Comprehensive Cancer Center Population Research Core, University of Iowa, Iowa City, IA 52242, USA; ^3^Holden Comprehensive Cancer Center Biostatistics Core, University of Iowa, Iowa City, IA 52242, USA; ^4^College of Public Health, Department of Epidemiology, University of Iowa, Iowa City, IA 52242, USA

## Abstract

*Objectives*. The study objective was to determine how insurance status relates to treatment receipt and overall survival for patients with early-stage pancreatic exocrine carcinoma.* Methods*. SEER data were evaluated for 17,234 patients diagnosed with Stage I/II pancreatic exocrine carcinoma. Multivariate regression models controlled for personal characteristics to determine whether insurance status was independently associated with overall survival and receipt of radiation/surgery.* Results*. Odds of receiving radiation were 1.50 and 1.75 times higher for insured patients compared to Medicaid and uninsured patients, respectively (*p* < 0.01). Insured patients had 1.68 and 1.57 times increased odds of receiving surgery compared to Medicaid and uninsured patients (*p* < 0.01). Risk of death was 1.33 times greater (*p* < 0.01) in Medicaid patients compared to insured patients; when further adjusted for treatment, the risk of death was attenuated but remained significant (HR = 1.16, *p* < 0.01). Risk of death was 1.16 times higher for uninsured patients compared to insured patients (*p* = 0.02); when further adjusted for treatment, the risk of death was no longer significant (HR = 1.01, *p* = 0.83).* Conclusions*. Uninsured and Medicaid-insured patients experience lower treatment rates compared to patients who have other insurances. The increased likelihood of treatment appears to explain the insured group's survival advantage.

## 1. Introduction

Average survival time for pancreatic exocrine carcinoma patients is among the poorest of all cancer types. Recent years have seen little improvement [1], with pancreatic cancer expected to become the second leading cause of cancer-related death by 2030 [2]. Although there are effective treatments for early-stage disease [3], many patients do not receive them. A minority of patients with early-stage disease undergo pancreatic resection, for example, even though it is a first-line therapy and is associated with improved survival [4–8]. Determining the reason for this could inform strategies to increase treatment rates and improve survival.

Disparities in insurance coverage may play an important role. It is known that patients with cancer at common sites (e.g., breast, colon, and lung) who lack insurance coverage have worse survival and inadequate treatment when compared to patients with insurance coverage [9]. Shapiro et al. used data from the Surveillance, Epidemiology, and End Results (SEER) program to establish that the same relationship exists for surgical treatment of pancreatic adenocarcinoma [10]. A SEER Patterns of Care study similarly showed that pancreatic cancer patients were more likely to receive cancer-directed surgery if they were insured [11]. Furthermore, two studies covering separate states have shown that patients with Medicaid or government-subsidized insurance have lower rates of cancer-directed surgery compared to those with private insurance [12, 13].

Disparities for those specifically covered by Medicaid have not been examined using the SEER dataset, the nation's only multistate, population-based cancer resource that contains comprehensive clinical and survival data [14]. The goal of our study was to use this resource to examine whether types of insurance are associated with treatment and survival disparities for pancreatic cancer patients.

## 2. Materials and Methods

Data from the 2016 release of the Surveillance, Epidemiology, and End Results (SEER) research dataset were used in this analysis [15]. The study cohort comprised 17,234 patients diagnosed with AJCC 6th Edition Stage IA/IB/IIA/IIB pancreatic exocrine carcinomas (ICD-O-3 site codes: C25.0, C25.1, and C25.2; histology codes: 8000, 8010, 8020, 8021, 8022, 8140, 8141, 8144, 8145, 8230, 8255, 8440, 8470, 8480, 8481, 8490, 8500, 8521, and 8560) between the years 2007 and 2013. Cases must have had known age/resection status and not be diagnosed at autopsy or by death certificate. Cases with unknown insurance status were also excluded, leaving the following SEER Insurance Recode categories for analysis: insured (which SEER created by combining private insurance, Medicare, other government insurance plans, and unspecified insurance), uninsured, and Medicaid coverage.

Logistic regression models were utilized to assess the association between insurance status and receipt of radiation or surgery (considered separately) while adjusting for age, sex, race, year of diagnosis, and stage. Estimated effects of predictors are reported as odds ratios (OR) along with 95% confidence intervals. Cox regression was used to determine whether insurance status was a prognostic indicator of overall survival (OS) while adjusting for age, sex, race, year of diagnosis, and stage. Time was calculated from date of diagnosis to all-cause death. Patients still alive at the end of 2013 were censored. Estimates of predictors are reported as hazard ratios (HR) along with 95% confidence intervals. All tests were two-sided and performed at the 5% significance level using SAS v9.4 (SAS Institute, Cary, NC).

## 3. Results

### 3.1. Insurance Status and Patient Characteristics

Patients' characteristics and disease variables are shown in Table 1. The majority of patients had Stage II disease (77.9%, *n* = 13,411). Most of the patients were white (81.8%, *n* = 14,090), and 51.1% of the patients were female (*n* = 8,799). Radiation therapy was provided to 30.4% of patients (*n* = 5,237), and 47.6% of patients underwent surgery at the primary cancer site (*n* = 8,210). Most patients were insured by private insurance, Medicare, or other government insurance plans (87.0%, *n* = 14,997); others were covered by Medicaid (10.8%; *n* = 1,860) or were uninsured (2.2%; *n* = 377).

To ensure that Medicare eligibility status did not affect the results, the interaction between age (<65 and 65+ years) and insurance status was examined in all models described in Table 1. This interaction between insurance status and age was found to be practically and statistically nonsignificant in all models. Thus, results from the more parsimonious, noninteraction models are reported Table 1.

### 3.2. Type of Insurance and Odds of Therapy

Table 2 illustrates the relationship between type of insurance and the odds of receiving radiation therapy or surgery after adjusting for age at diagnosis, year of diagnosis, race, sex, and stage. Insured patients had 1.50 and 1.75 times increased odds of receiving radiation compared to Medicaid and uninsured patients, respectively (both *p* < 0.01). Similarly, insured patients were more likely to undergo surgery compared to Medicaid and uninsured patients (odds ratios of 1.68 and 1.57, resp.; both *p* < 0.01). There were no significant differences between the uninsured patients and those with Medicaid coverage for either radiation or surgical treatment.

### 3.3. Multivariate Survival Analysis

Table 3 shows survival as a function of insurance status adjusted for age at diagnosis, year of diagnosis, race, sex, and stage. The risk of death was 1.33 times greater (*p* < 0.01) in Medicaid patients compared to insured patients. Uninsured patients were at a higher risk of death compared to insured patients (HR = 1.16, *p* = 0.02). A borderline significant (*p* = 0.05) finding was that Medicaid patients had poorer overall survival compared to uninsured patients (HR = 1.14, *p* = 0.05).

Table 3 also shows the results of a multivariate analysis relating survival to insurance status when further adjusted for receipt of radiation and/or surgery. The result was an attenuation of some of the HRs shown in Table 3 with risk of death reduced to 1.16 times greater (*p* < 0.01) in Medicaid patients compared to insured patients, with no indication of a survival benefit for uninsured versus insured patients (HR = 1.01, *p* = 0.83). Once again, a borderline significant (*p* = 0.05) finding was that Medicaid patients had poorer overall survival compared to uninsured patients (HR = 1.14, *p* = 0.05).

## 4. Discussion

Pancreatic exocrine carcinomas are aggressive and deadly, but proven treatments exist for early-stage disease. Disparities in delivery of these curative or life-prolonging therapies present a major obstacle to improving patient survival [16]. We observed that uninsured and Medicaid-insured pancreatic exocrine carcinoma patients experience lower odds of receiving both radiation therapy and surgical resection compared to patients who have other insurances. We also observed no difference for receipt of resection or radiation between noninsured and Medicaid-insured patients. These findings have implications for the recent efforts to expand Medicaid programs with the aim to increase access to care [17], even though it is not necessarily true that expanding these programs as they currently exist will have the desired effect.

We also observed a relationship between insurance status and overall survival for pancreatic exocrine carcinoma. This relationship has been established for other types of cancers [9, 18–22]. Our analysis further revealed that both the Medicaid and uninsured populations had a significantly higher risk of death than the insured population after controlling for other individual and disease factors.

It is especially interesting that the survival difference between the insured and the uninsured populations is absent when the analysis is controlled for receipt of therapy (HR = 1.01). This suggests that improved survival for insured patients may exist because they are more likely to receive therapy. However, something different is observed for the Medicaid-insured population: these patients have poorer survival even after controlling for receipt of resection and radiation. This suggests that worse outcomes for this group are due to more than just lack of treatment. One explanation for poorer survival for Medicaid patients is that they may have poorer health status overall. Medicaid patients may also lack the provider options that privately insured patients have access to, and this could delay care or compromise expertise. Patients with Medicaid insurance or without insurance might experience less treatment or less aggressive care in other ways, which diminishes their survival potential, including less adherence to treatment guidelines [23, 24].

Unfortunately, the SEER research dataset does not include comorbidity data and other variables that may allow us to explore these possibilities, and this represents a limitation of the study. Another limitation of the SEER data is the lack of drug-specific chemotherapy data. Also, the relatively small sample size for the uninsured group could have affected the power of some of the analyses. An additional limitation is that the Insurance Recode variable provided in the SEER research dataset combines several insurance categories (i.e., private insurance, Medicare, other government insurance plans, and unspecified insurance), and it is possible that these subtypes have different associations with treatment and survival. A strength of the study is that it employs a very large cohort of patients from high-quality SEER registries that cover approximately 28% of the population of the United States [14].

Addressing insurance disparities may improve aggregate survival and individual patient outcomes using proven therapies. Specifically, increasing rates of pancreatectomy will likely increase the average survival of pancreatic exocrine carcinoma patients [4, 21]. Greater adherence to accepted guidelines offers patients and providers the opportunities for improved outcomes [24]. Increased education among providers about the indications for treatment and improvements in survival is needed. Additionally, improved safeguards should be implemented to minimize the influence of nonclinical factors, including insurance status, on treatment planning decisions.

## Figures and Tables

**Table 1:** Patients' characteristics by insurance status.

Characteristic	Level	Any Medicaid *N* = 1860 *N* (%)	Insured^1^ *N* = 14997 *N* (%)	Uninsured *N* = 377 *N* (%)	Total	%
Site	C25.0-head of pancreas	1573 (84.6)	12259 (81.7)	319 (84.6)	14151	82.1
	C25.1-body of pancreas	147 (7.9)	1457 (9.7)	34 (9.0)	1638	9.5
	C25.2-tail of pancreas	140 (7.5)	1281 (8.5)	24 (6.4)	1445	8.4

Age	<40	20 (1.1)	85 (0.6)	10 (2.7)	115	0.7
	40–49	132 (7.1)	519 (3.5)	54 (14.3)	705	4.1
	50–59	429 (23.1)	1968 (13.1)	125 (33.2)	2522	14.6
	60–69	462 (24.8)	4026 (26.8)	129 (34.2)	4617	26.8
	70–79	453 (24.4)	4573 (30.5)	31 (8.2)	5057	29.3
	80+	364 (19.6)	3826 (25.5)	28 (7.4)	4218	24.5

Sex	Female	1053 (56.6)	7557 (50.4)	189 (50.1)	8799	51.1
	Male	807 (43.4)	7440 (49.6)	188 (49.9)	8435	48.9

Race	Black	352 (18.9)	1465 (9.8)	69 (18.3)	1886	10.9
	Other	243 (13.1)	980 (6.5)	35 (9.3)	1258	7.3
	White	1265 (68.0)	12552 (83.7)	273 (72.4)	14090	81.8

Year of diagnosis	2007–2010	981 (52.7)	8040 (53.6)	184 (48.8)	9205	53.4
	2011–2013	879 (47.3)	6957 (46.4)	193 (51.2)	8029	46.6

Stage	IA/IB	460 (24.7)	3297 (22.0)	66 (17.5)	3823	22.2
	IIA	681 (36.6)	5077 (33.9)	147 (39.0)	5905	34.3
	IIB	719 (38.7)	6623 (44.2)	164 (43.5)	7506	43.6

Radiation	No	1393 (74.9)	10329 (68.9)	275 (72.9)	11997	69.6
	Yes	467 (25.1)	4668 (31.1)	102 (27.1)	5237	30.4

Surgery	No	1147 (61.7)	7676 (51.2)	201 (53.3)	9024	52.4
	Yes	713 (38.3)	7321 (48.8)	176 (46.7)	8210	47.6

Vital status at end of 2013	Alive	426 (22.9)	4065 (27.1)	132 (35.0)	4623	26.8
	Deceased	1434 (77.1)	10932 (72.9)	245 (65.0)	12611	73.2

Mean age at diagnosis (SD)		67.0 (12.9)	70.8 (11.6)	59.8 (11.5)		

^1^Insured status was created by SEER to include private insurance, Medicare, other government insurance plans, and unspecified insurance.

**Table 2:** Adjusted odds ratios for receiving radiation or surgery by insurance status.

Outcome	Comparison		OR	95% CI		*P* value
Odds of receiving radiation^1^	Insured	Versus any Medicaid	1.50	1.34	1.68	<**.01**
	Uninsured	Versus any Medicaid	0.85	0.66	1.10	0.23
	Insured	Versus uninsured	1.75	1.39	2.22	<**.01**

Odds of undergoing surgery^1^	Insured	Versus any Medicaid	1.68	1.50	1.88	<**.01**
	Uninsured	Versus any Medicaid	1.07	0.84	1.37	0.58
	Insured	Versus uninsured	1.57	1.25	1.97	<**.01**

^1^Adjusted for age at diagnosis, sex, year of diagnosis, race, and stage.

**Table 3:** Adjusted hazard ratios for all-cause survival by insurance status.

Outcome	Comparison		Controlled for personal characteristics				Controlled for personal characteristics and treatment received			
			HR^1^	95% CI		*P* value	HR^2^	95% CI		*P* value
Overall survival	Any Medicaid	Versus insured	1.33	1.25	1.40	<**.01**	1.16	1.10	1.23	<**.01**
	Any Medicaid	Versus uninsured	1.14	1.00	1.31	0.05	1.14	1.00	1.31	0.05
	Uninsured	Versus insured	1.16	1.02	1.32	**0.02**	1.01	0.89	1.15	0.83

^1^Adjusted for age at diagnosis, sex, year of diagnosis, race, and stage.

^2^Adjusted for age at diagnosis, sex, year of diagnosis, race, stage, radiation, and/or surgery.
